# Diffusion-weighted MRI in the identification of renal parenchymal involvement in children with a first episode of febrile urinary tract infection

**DOI:** 10.3389/fradi.2024.1452902

**Published:** 2024-11-21

**Authors:** Lorenzo Anfigeno, Alberto La Valle, Elio Castagnola, Enrico Eugenio Verrina, Giorgio Piaggio, Maria Ludovica Degl'Innocenti, Emanuela Piccotti, Andrea Wolfler, Francesca Maria Lembo, Monica Bodria, Clelia Formigoni, Alice Boetto, Lucia Santini, Maria Beatrice Damasio

**Affiliations:** ^1^ Department of Radiology, Giannina Gaslini Institute (IRCCS), Genoa, Italy; ^2^ Infectious Disease Unit, Giannina Gaslini Institute (IRCCS), Genoa, Italy; ^3^ Department of Nephrology and Kidney Transplantation, Giannina Gaslini Institute (IRCCS), Genoa, Italy; ^4^ Department of Emergency Pediatrics and First Aid, Giannina Gaslini Institute (IRCCS), Genoa, Italy; ^5^ Department of Anesthesiology and Acute and Procedural Pain Therapy, Giannina Gaslini Institute (IRCCS), Genoa, Italy; ^6^ Ausl Parma, Dipartimento Cure Primarie, Distretto Sud-Est, Parma, Italy; ^7^ Department of Health Sciences, School of Medical and Pharmaceutical Sciences, University of Genoa, Genoa, Italy

**Keywords:** UTI (urinary tract infection), pyelonephritis, pediatric, DWI (diffusion weighted imaging), MRI

## Abstract

**Aims:**

This study aims to assess the diagnostic accuracy of diffusion-weighted Magnetic Resonance Imaging (DW-MRI) and determine the inter-reader agreement between two expert radiologists in detecting pyelonephritic foci during the initial episode of febrile urinary tract infection (fUTI) in children aged 0–5 years. Also, we aim to establish the correlation between clinical data and DW-MRI findings.

**Methods:**

Children aged 0–5 years presenting with their first episode of fUTI were included in the study and underwent DW-MRI and Ultrasound (US) examinations within 72 h of admission. Inter-observer agreement between the two expert radiologists in assessing DW-MRI scans was evaluated using Cohen's kappa statistic. Clinical and laboratory data were subjected to statistical analysis.

**Results:**

84 children (40 male, 44 female) with a mean age of 7.3 (SD 6.2) months were enrolled. DW-MRI detected pyelonephritis in 78 out of 84 cases (92.9%), with multiple foci observed in 73 out of 78 cases (93.6%). There was a “substantial” level of agreement between the two expert radiologists (*κ* = 0.725; observed agreement 95.2%). Renal US revealed pyelonephritis in 36 out of 78 cases (46.2%). White blood cell (WBC) count (*p* = 0.04) and lymphocyte count (*p* = 0.01) were significantly higher in patients with positive DW-MRI. Although not statistically significant, patients with positive DW-MRI had higher mean values of C-Reactive Protein, Procalcitonin, and neutrophil WBC count (7.72 mg/dl, 4.25 ng/dl, and 9,271 /μl, respectively).

**Conclusions:**

DW-MRI exhibited excellent diagnostic performance in detecting pyelonephritic foci, with substantial inter-reader agreement among expert radiologists, indicating the reliability of the technique. However, a weak correlation was observed between laboratory parameters and DW-MRI results, potentially because of the low rate of negative DW-MRI findings.

## Introduction

Urinary tract infections (UTIs) represent the most prevalent bacterial infections in pediatric populations. Within the first year of life, UTIs affect approximately 0.7% of girls and 2.7% of uncircumcised boys (1). During the initial 6 months, uncircumcised boys exhibit a 10–12-fold higher risk of UTI compared to girls, while beyond the first year, females are more commonly affected (1).

Infants with febrile UTI (fUTI) may manifest nonspecific symptoms, including fever, irritability, lethargy, poor feeding, seizures, and cyanosis (1). Acute pyelonephritis (APN) is diagnosed in the presence of fever (≥38°C) and positive urine culture (2).

A urine dipstick test, a rapid and cost-effective diagnostic tool, can serve as an initial screening test for fUTIs in children, pending urine culture results (3). Key parameters to assess include leukocyte esterase (LE), which exhibits high sensitivity (up to 94%) but lower specificity (approximately 72%), and nitrites (3, 4). Although the nitrite test demonstrates low sensitivity, particularly in infants, because of the time required for bacterial conversion of nitrates introduced through diet (approximately 4 h), it boasts high specificity (98%). Therefore, a positive nitrite test strongly suggests a UTI diagnosis (4).

A recognized risk factor for the development of acute pyelonephritis (APN) is vesicoureteral reflux (VUR), particularly when of high grade (5). Voiding cystourethrography (VCUG) stands as the gold standard for VUR detection (5, 6), although contrast-enhanced voiding urosonography (ceVUS) has emerged as a non-radiating alternative technique (7, 8).

In the clinical literature, elevated levels of C-reactive protein (CRP), white blood cells (WBC), and absolute neutrophil count (ANC) are indicative of APN but lack specificity and sensitivity in distinguishing it from lower tract UTIs (1, 9). Procalcitonin (PCT) exhibits higher sensitivity and specificity for APNs and may predict renal scarring development (1, 10), though further studies are warranted to confirm its significance (9).

While color-Doppler ultrasound (cd-US) demonstrates limited sensitivity in detecting parenchymal lesions, it remains crucial for identifying anatomical predisposing factors such as ureteral anomalies, calyceal-pelvic-ureteral dilatation, and bladder anomalies (2, 3, 11). Dimercaptosuccinic acid (DMSA) scintigraphy remains the gold standard for detecting parenchymal involvement in APN patients (12). Contrast-enhanced computed tomography (CT) is also highly sensitive and specific for identifying APNs, though concerns persist regarding radiation exposure and contrast medium administration (13).

Diffusion-weighted Magnetic Resonance Imaging (DW-MRI) emerges as a promising, non-radiating, and contrast-medium-free imaging modality for diagnosing acute pyelonephritis (APN) in children with febrile urinary tract infection (fUTI). Literature reports higher sensitivity and specificity of DW-MRI compared to DMSA scintigraphy, with no need for contrast medium injection or radiation exposure (14). Also, DW-MRI exhibits excellent agreement (*κ* = 0.92) with gadolinium-enhanced T1W-MRI findings in APN (15). The examination duration is brief; however, mild sedation may be necessary for uncooperative children.

In DW-MRI, renal inflammatory lesions manifest as areas of restricted diffusion, appearing hyperintense on DW images and hypointense on corresponding apparent diffusion coefficient (ADC) maps (16).

According to EAU guidelines, US is recommended for all patients presenting to the emergency department with febrile urinary tract infections (fUTI) (17). According to current EAU guidelines, both DW-MRI and DMSA scintigraphy can confirm APN during the acute phase (18).

This study aims to explore the diagnostic role of DW-MRI in identifying renal parenchymal involvement during the initial episode of fUTI in children aged 0–5 years with no prior history of fUTI. Also, the study seeks to evaluate the inter-observer agreement between two expert radiologists in blind MRI interpretation and correlate DW-MRI findings with clinical and laboratory data. A secondary objective is to compare the sensitivity and specificity of color-Doppler ultrasound (US) vs. DW-MRI in identifying renal parenchymal involvement.

## Materials and methods

This cross-sectional observational study enrolled patients aged ≤5 years admitted to our institute between January 2022 and January 2023 diagnosed with febrile urinary tract infection (fUTI), defined by a body temperature ≥38°C, positive urine culture, and abnormal urinary dipstick results. A positive urine dipstick was characterized by positive leukocyte esterase (LE) alone, or both LE and red blood cells (RBC), or a combination of LE, RBC, and nitrites. Each patient underwent ultrasound (US) and diffusion-weighted magnetic resonance imaging (DW-MRI) within 72 h of admission.

### Inclusion criteria

Patients aged ≤5 years with a diagnosis of a first episode of fUTI, defined as body temperature ≥38°C and:

• Age ≤5 years
• Diagnosis of a first episode of febrile urinary tract infection (fUTI), defined as a body temperature ≥38°C and:
• Positive urine culture [≥100,000 colony-forming units (CFU)/ml of a single species of uropathogenic] and positive urine dipstick, or urine culture with mixed flora and positive urine dipstick, accompanied by clinical and laboratory parameters suggestive of acute pyelonephritis (APN) (e.g., elevated white blood cell [WBC] count, absolute neutrophil count [ANC], inflammatory markers; presence of fever, urinary symptoms; positive ultrasound [US] findings)
• Positive urine dipstick and negative urine culture when obtained after the initiation of antibiotic therapy.

### Exclusion criteria

• Age >5 years
• History of previous episodes of febrile urinary tract infection (fUTI)
• Body temperature <38°C
• Severe pelvic dilatation (anterior-posterior diameter >20 mm)
• Severe renal hypoplasia and/or impaired renal function [estimated glomerular filtration rate (eGFR) <70 ml/min/1.73 m^2^ using the Schwartz formula] (21)
• Combination of negative urine culture and negative urine dipstick
• Transplanted kidneys

The clinical and laboratory data of the enrolled patients included:

1. Axillary temperature recorded upon admission.
2. Duration of fever from onset at home until presentation, measured 6 h after the last administration of acetaminophen.
3. Symptoms observed upon admission.
4. Results of urinary dipstick analysis, including assessing chemical and physical parameters such as urinary pH, presence of nitrites, leukocyte esterase (LE), and red blood cells (RBC).
5. Urine culture results and antibiogram of the causative microorganism of urinary tract infection (UTI).
6. Blood test parameters, encompassing C-reactive protein (CRP) and procalcitonin (PCT) levels, white blood cell (WBC) count, absolute neutrophil count (ANC), lymphocyte count, platelet (PLT) count, creatinine, blood urea nitrogen (BUN), and estimated glomerular filtration rate (eGFR). The nephrology laboratory of our institute established normal values that varied based on the children's ages.

In uncollaborative children a combination of intranasal dexmedetomidine and midazolam was the first choice; only in case of unsuccess, inhalational (sevoflurane in spontaneous breathing with facial mask) or intravenous (continuous infusion of propofol) sedation was performed. All children were monitored with SpO2 and heart rate during the exam.

All MRI examinations were performed using a 1.5 T scanner (Philips© Intera Achieva 1.5 T, release 5) equipped with a 32-channel cardiac or body coil, or a pediatric 8-channel coil. See Table 1 with MRI-protocol. Two expert radiologists, each with at least 6 years of experience in assessing renal diffusion-weighted imaging (DWI) scans, independently evaluated the MRI scans.

**Table 1:** MRI protocol.

Sequence	Plane	Slice thickness	GAP	TR	TE	NSA	B value
Single shot T2	Axial	4mm	0,4	shortest	80	2	
Single shot T2	Coronal	4mm	0	shortest	80	1	
TSE T2 HR-RT	Coronal	4mm	0,4	shortest	100	2	
DWI	Axial	4mm	0,4	shortest	69	3	0, 50, 300, 600, 1,000
DWI	Coronal	4mm	0,4	shortest	69	3	0, 50, 300, 600, 1,000

In the DWI sequence, we evaluated the following parameters:

• Identification of focal parenchymal areas demonstrating restricted diffusion.
• Localization, number, and unilateral or bilateral presence of focal renal lesions.
• We documented the corresponding focal signal alterations on the T2-weighted sequence.

Abnormal ultrasound (US) findings considered were:

• Focal alterations in renal parenchymal echotexture.
• Poor corticomedullary differentiation.
• Focal hypoperfusion detected by color Doppler.
• Mild pelvicalyceal dilatation [>10 mm; <20 mm, according to UTD classification (20)] and/or ureteral dilatation.
• Thickening of the pelvic walls.

Patients with fUTI and abnormal US also performed ceVUS or VCUG as reflux test.

The primary outcome of this study was to assess the diagnostic performance of diffusion-weighted MRI (DW-MRI) in identifying renal parenchymal involvement in patients aged 0–5 years experiencing their first episode of febrile urinary tract infection (fUTI).

Secondary outcomes included evaluating the inter-observer agreement between two expert radiologists in blind interpretation of MRI scans, correlating DW-MRI findings with clinical and laboratory data, and investigating the sensitivity and specificity of color-Doppler ultrasound (US) compared to DW-MRI in detecting renal parenchymal involvement.

### Statistical analysis

Descriptive statistics were computed for the entire cohort, with continuous variables expressed as mean and standard deviation, while median value and range were calculated and reported. Absolute or relative frequencies were provided for categorical variables.

Differences between groups were assessed using non-parametric analysis (Mann–Whitney *U*-test) for continuous variables and Chi-square or Fisher's exact test for categorical variables.

Inter-observer agreement was determined using Cohen's kappa (*κ*), with interpretation based on predefined cut-offs: <0 (poor), 0.00–0.20 (slight), 0.21–0.40 (fair), 0.41–0.60 (moderate), 0.61–0.80 (substantial), 0.81–1 (almost perfect) (21).

We considered a *p*-value less than 0.05 statistically significant and conducted all tests as two-tailed. Statistical analysis was performed using SPSS for Windows (SPSS Inc., Chicago, Illinois, USA).

## Results

### Sample description

Our sample comprised 84 patients experiencing their initial episode of febrile urinary tract infection (fUTI): 40 out of 84 (47.6%) patients were male, while 44 out of 84 (52.4%) were female. The mean age at presentation was 7.3 (SD 6.2) months (refer to Table 2 for demographic characteristics).

**Table 2:** Demographic characteristics of patients involved.

*N* = 84 (40 Male; 44 Female)	DWI negative	DWI positive	*P*_value
	*N* = 6	*N* = 78	
	*N* (%)	*N* (%)	
Gender, M	3 (50)	37 (47.4)	1
	Mean (SD)	Mean (SD)	
Age (months)	6.4 (1.6)	7.4 (6.4)	0.77
Admission-DWI (days)	1.67 (0.8)	2.08 (1.1)	0.41
Height (cm)	70.5 (6.4)	68.7 (10.9)	0.15
Weight (kg)	7.5 (1,4)	7.9 (2.8)	0.98

### Imaging results

**Figure 1:**
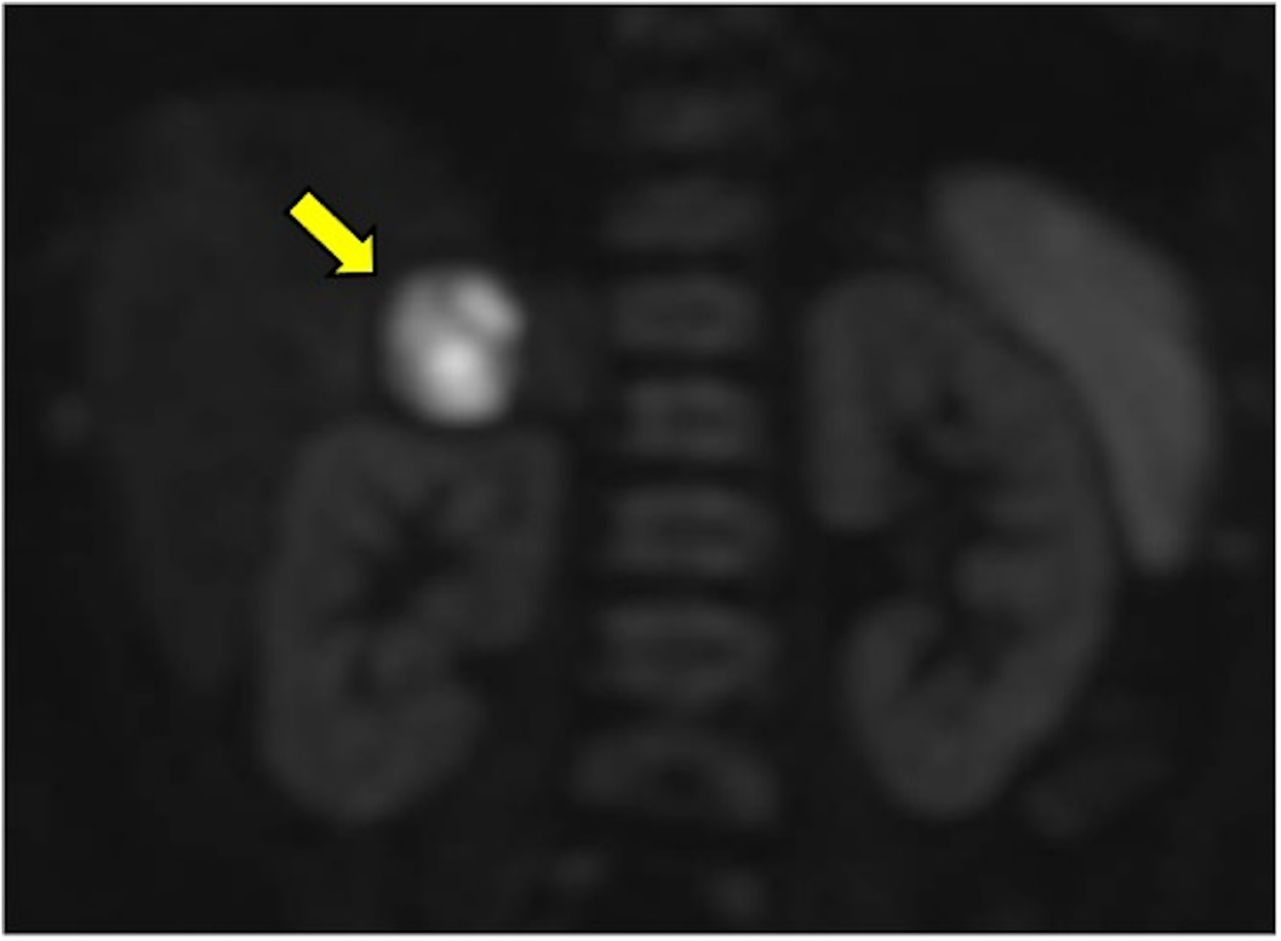
A 6-month-old child with fUTI; DW-MRI showed no focal renal areas of restricted diffusion. Right adrenal hemorrhage (arrow).

In the DW-MRI assessment, findings were positive in 78 out of 84 patients (92.9%), while 6 out of 84 (7.1%) showed negative results (Figure 1). Among the positive cases, bilateral involvement was noted in 46 out of 78 patients (59%), with the right kidney affected in 61 out of 78 cases (78.2%) and the left kidney in 59 out of 78 cases (75.6%). Specifically, DW-MRI revealed a single renal area with restricted diffusion in 5 of 78 cases (6.4%), while we observed multiple renal foci in 73 of 78 cases (93.6%). Further details regarding the distribution of renal parenchymal foci are presented in Table 3.

**Table 3:** DW-MRI results.

DWI	*N* (%)
DWI positive	78/84 (92.9%)
DWI negative	6/84 (7.1%)
Kidney involved: right	24/78 (30.8%)
Left	22/78 (28.2%)
Both	46/78 (59%)
Number of pyelonephritic areas	
One	5/78 (6.4%)
Multiple	73/78 (93.6%)

We observed a positive correlation between T2-weighted MRI (T2-MRI) and diffusion-weighted MRI (DW-MRI) findings, with focal signal alterations occurring in the same area, in 50 out of 84 patients (59.5%).

Urinary tract ultrasound (US) was performed in all 84 patients (100%). Positive findings were noted in 36 out of 84 patients (46.2%), all of whom also had positive DWI results. Specifically, bilateral positive findings were present in 10 out of 78 patients (12.8%), positive findings in the left kidney were observed in 14 out of 78 patients (17.9%), and positive findings in the right kidney were found in 12 out of 78 patients (15.4%). In the remaining 42 out of 78 cases (53.8%), US findings were negative for acute pyelonephritis (APN). The sensitivity of US compared to DWI was 46.2%, with a specificity of 100%. Pelvic dilatation was detected in 12 out of 78 patients (15.4%).

ceVUS or VCUG as reflux tests were performed in 33 patients.

11/33 patients (33.33%) resulted positive for vesico-ureteral reflux (VUR). All these 11 patients had positive DWI.

### Inter-observer concordance

The inter-reader agreement in detecting renal parenchymal acute pyelonephritis (APN) with DW-MRI between two qualified radiologists was substantial (Kappa 0.725, 95% CI 0.472; 0.978; Observed Agreement 95.2%). This result was further corroborated when considering the involvement of the right or left kidney separately: for the right kidney, the agreement was substantial (Kappa 0.760, 95% CI 0.617; 0.903; Observed Agreement 89.3%), and for the left kidney, it remained substantial (Kappa 0.782, 95% CI 0.639; 0.925; Observed Agreement 90.5%).

### Clinical presentation

Patients with positive DW-MRI findings exhibited a lower temperature at presentation compared to those with negative DWI (38.9°C (SD 0.7) vs. 39.5°C (SD 0.5); *p* = 0.05). Also, although not statistically significant, patients with positive DWI had a longer duration of fever (85.8 (SD 58.7) vs. 44.3 (SD 29.6) hours; *p* = 0.06).

The mean time between the onset of fever and DW-MRI was 3.74 (SD 2.1) days.

The most common symptoms associated with fever were feeding difficulties (27/78, 35.1%), diarrhea (17/78, 22.1%), cloudy and foul-smelling urine (13/78, 15.9%), vomiting (11/78, 14.3%), reduced activity (7/78, 9.1%), convulsions, or neurological symptoms such as loss of consciousness (7/78, 9.1%). However, no statistically significant relationship was found between clinical presentation and DWI results.

Also, there were no statistically significant differences in age, weight, length, or gender between patients with positive or negative DWI findings at the presentation of febrile urinary tract infection (fUTI) (see Table 4).

**Table 4:** Clinical manifestations.

	DWI−	DWI+	*P*_value
	*N* = 6	*N* = 78	
	*N* (%)	*N* (%)	
Irritability, *yes*	1 (20)	13 (16.9)	1
Low feeding, *yes*	3 (60)	27 (35.1)	0.35
Vomiting, *yes*	1 (20)	11 (14.3)	0.56
Dhiarrea, *yes*	2 (40)	17 (22.1)	0.33
Lethargy, *yes*	1 (20)	7 (9.1)	0.41
Foul smelly urines, *yes*	1 (20)	13 (15.9)	1
Convulsions, *yes*	1 (20)	7 (9.1)	0.41
	Mean (SD)	Mean (SD)	
BT (°C) at admission	39.48 (0.46)	38.94 (0.73)	0.05
Hours of fever	44.33 (29.63)	85.47 (58.67)	0.06

### Urinary data

We conducted a urinary dipstick analysis in 83 out of 84 patients (98.8%), with 1 patient (1.2%) having undergone a positive complete urine examination (including sediment analysis) before admission to the emergency room. Among the 83 patients, the dipstick showed a combination of nitrites, leukocyte esterase (LE), and red blood cells (RBC) in 56 patients (67.5%), a combination of LE and RBC in 19 patients (22.9%), and positivity only for LE in 8 patients (9.6%). Also, 42 out of 83 (50.6%) patients underwent repeat urinary dipstick testing, revealing a combination of nitrites, LE, and RBC in 19 out of 42 (45.2%), positivity for a combination of LE and RBC in 10 out of 42 (23.8%), and positivity only for LE in 8 out of 42 (19%) cases (refer to Table 5).

**Table 5:** Urinary stick results and blood test.

	DWI negative	DWI positive	*P*_value
	*N* = 6	*N* = 78	
	Mean (SD)	Mean (SD)	
Stick 1			
Specific gravity	1,013.3 (7.5)	1,012.2 (6)	0.67
pH	5.7 (0.8)	5.7 (0.8)	0.81
Leukocytes	2.5 (0.5)	2.8 (0.6)	0.11
Nitrites	1.0 (1.1)	0.8 (0.8)	0.87
Erythrocytes	1.8 (1.5)	1.9 (1.4)	0.80
Blood test			
WBC × 10^3^ /ul	10,531 (3,063)	15,736 (5,944)	0.04
Neutrophils	6,553 (2,847)	9,270 (4,843)	0.16
Lymphocytes	2,626 (842)	4,477 (1,976)	0.01
Platelets	3,39,333 (1,04,840)	4,27,961 (1,37,085)	0.09
PCR (mg/dl)	3.9 (4.8)	7.7 (6.7)	0.08
PCT (ng/ml)	2.3 (4.7)	4.2 (13.8)	0.33
Creatinine (mg/dl)	0.25 (0.03)	0.25 (0.05)	0.91
eGFR sec Schwartz (ml/min)	118.4 (13.7)	116.1 (27.9)	0.51
BUN (mg/dl)	15.3 (4.3)	18.9 (12.4)	0.49

We performed a urine culture in all 84 patients (100%), with 78 out of 84 cultures (92.9%) conducted before the initiation of antibiotic therapy. The remaining 6 cultures (7.1%) were negative, as they were conducted after antibiotic administration, despite all six patients having positive urinary dipstick tests. Among the positive cultures, a single bacterium was identified in 75 out of 84 cases (89.3%), with Escherichia coli (E. coli) being the most frequently isolated microorganism (68/75, 90.7%). In 7 out of 75 cases (9.33%), other bacterial species were detected. Also, 3 out of 78 urine cultures (3.85%) exhibited mixed flora with both gram-positive and gram-negative bacteria, along with pathological urinary dipstick results, fever, and symptoms suggestive of urinary tract infection (UTI).

### Blood test

All patients underwent blood tests before the initiation of antibiotic therapy. Patients with positive DW-MRI findings exhibited higher values of white blood cells (WBC) and lymphocytes compared to those with negative DWI (15,736.3 (SD 5,944.3) vs. 10,531.7 (SD 3,063.4); *p* = 0.04; 4,477.69 (SD 1,976.42) vs. 2,626.67 (SD 842.37); *p* = 0.01, respectively). However, the other parameters did not show significant differences between the two groups (refer to Table 5).

## Discussion

Building upon the established correlation between positive diffusion-weighted imaging (DWI) findings and APN (14, 15, 22), this study aims to assess the diagnostic accuracy of DW-MRI in identifying renal parenchymal involvement during the first episode of febrile urinary tract infection (fUTI) in children aged 0–5 years.

Also, it seeks to verify the inter-reader agreement between two expert radiologists in MRI evaluations and establish the relationship between clinical/laboratory parameters and MRI results.

The potential diagnostic role of DW-MRI in identifying renal parenchymal involvement in fUTI in children was already studied by our research group in 2021 (23). The patient cohort included in this study is homogeneous in age (0–5 years) when compared to previously published data, and we only recruited patients with a confirmed first episode of fUTI, ruling out any prior renal damage. We selected this age range to minimize the risk of previous UTI events, extending it to 5 years to ensure an adequate number of recruited patients.

We decided to include exclusion criteria such as severe renal hypoplasia and/or impaired renal function to ensure the kidneys were not significantly altered, as this would have been a confounding factor, and severe pelvic dilatation (>20 mm) due to limited assessable parenchyma, compressed by the dilatation.

The positive rate of diffusion-weighted MRI (DW-MRI) in our study was approximately 93%, a finding consistent with the existing literature (14). However, it contrasts with the results of our previous study, where the percentage was notably lower (23). We attribute this difference to the stricter patient inclusion criteria employed in the current study, which focused on patients experiencing their first febrile urinary tract infection (fUTI) within a narrower age range.

Moreover, the agreement between the two expert radiologists, specialized in uro-nephron radiology, was substantial (*κ* 0.725; observed agreement 95.2%), indicating the reliability of the technique.

**Figure 2:**
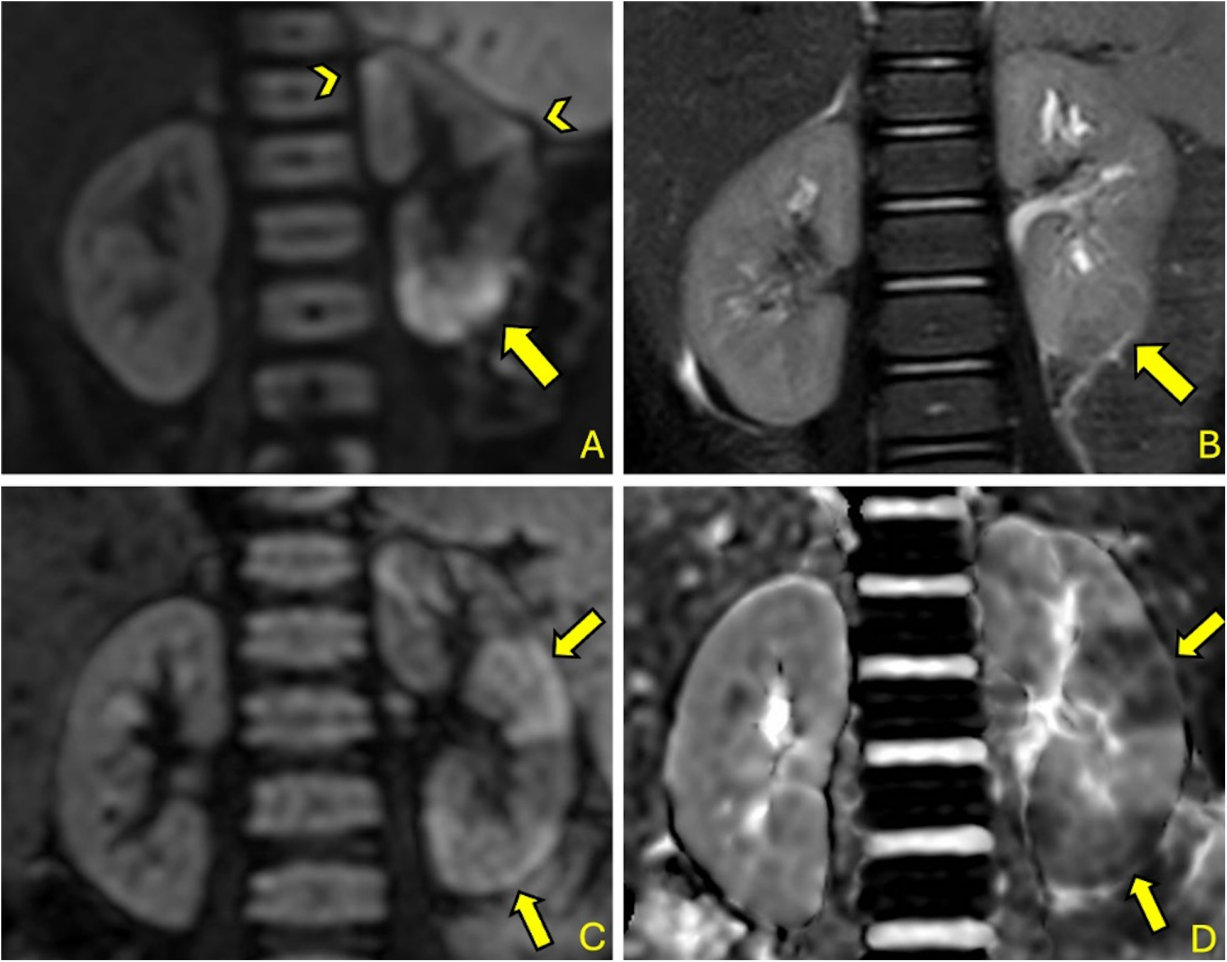
2-year-old girl with fUTI (A&B): DW-MRI (b = 1,000) **(A)** shows a significant focal area of restricted diffusion at left lower pole (arrow) with corresponding T2 signal alteration (arrow) **(B)**; other smaller foci are pointed by arrowhead **(A)** 35-month-old boy with fUTI (A&B): DW-MRI (b = 1,000) shows multiple focal areas of restricted diffusion (arrows) **(C)** with corresponding hypointensities in the ADC map (arrows) **(D)**.

DW-MRI, besides revealing a high positivity rate, demonstrated its spatial resolution and ability to precisely localize individual foci by revealing multiple foci (see Figure 2) in a significant proportion of cases. This capability could be extremely useful in follow-up assessments aimed at determining the relationship between acute foci and the potential development of scar complications.

Also, MRI produced positive results in a significant percentage of cases (59%), including T2-weighted sequences (refer to Figure 2), albeit with lower sensitivity than DWI.

**Figure 3:**
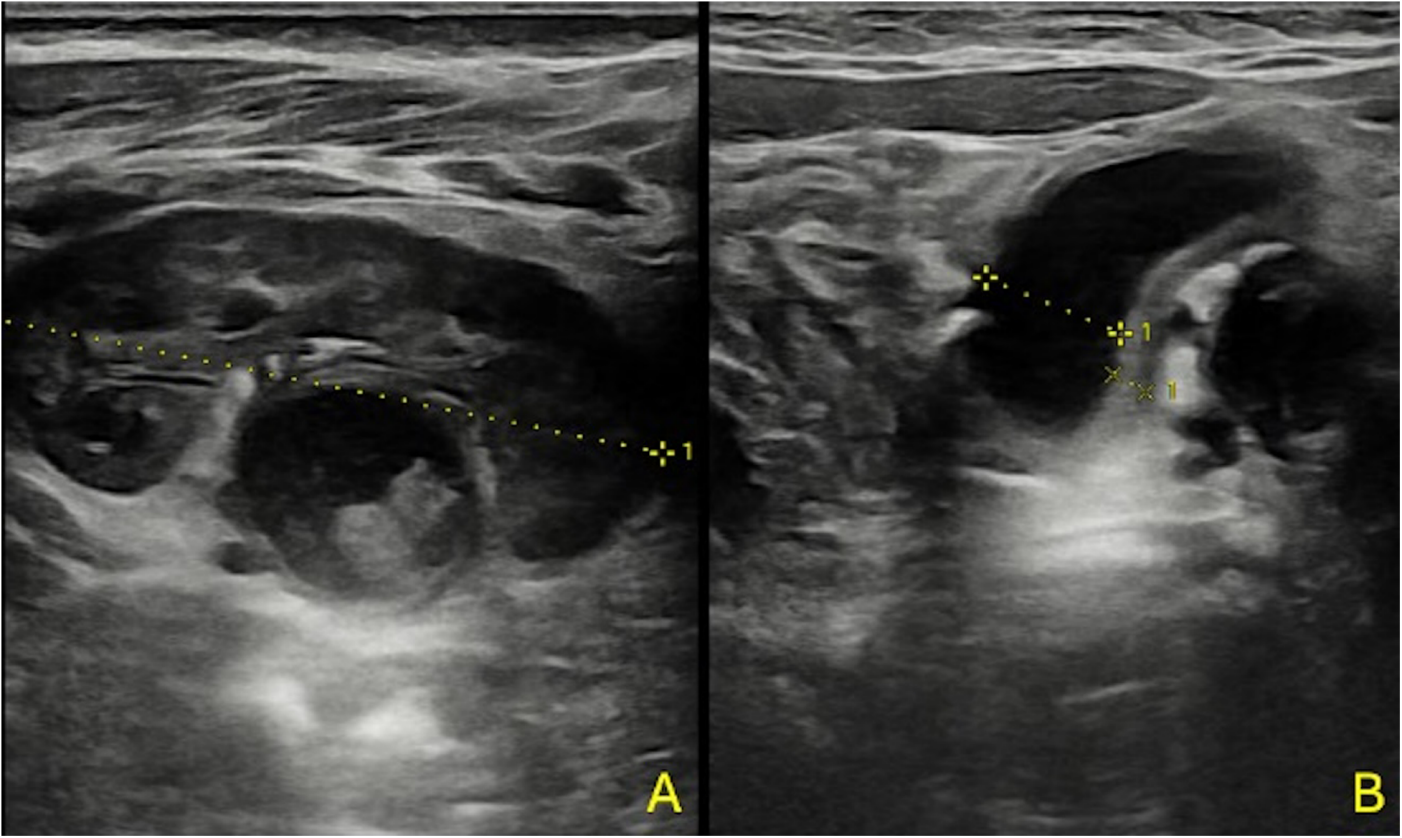
A 3-month-old boy with fUTI **(A,B)**; US shows no focal areas suggestive of APN, pelvic dilatation with thickened pelvic walls, and some echoic debris within the pelvis.

Color-Doppler ultrasound (cd-US) exhibited limited effectiveness in delineating renal foci, underscoring its primary role in identifying potential predisposing factors for acute pyelonephritis (APN) or associated findings (refer to Figure 3) (24).

Data from the literature suggest a superiority of contrast-enhanced ultrasound (CEUS) compared to conventional ultrasound (cd-US) (25–29). However, we cannot confirm this statement at the moment, as our data are still under analysis. This topic will be the subject of a future paper.

The rationale for utilizing DW-MRI in identifying renal parenchymal involvement during fUTI is to analyze the type of involvement (single or multiple foci, unilateral or bilateral, location) in relation to clinical data, especially since we do not precisely know the percentage of lesions in this age group that complicate into renal scarring, as well as the type of lesions that are more likely to cause scarring.

Literature indicates that recurrent fUTIs can lead to scarring complications, particularly when associated with VUR, potentially resulting in reflux nephropathy with chronic renal impairment (30–34).

We believe it is useful to identify the primary risk factors that can be studied using DW-MRI for the development of renal damage.

To make this investigation as non-invasive as possible, we have developed an MRI protocol with the shortest possible duration (approximately 5 min of scan time), based on rapid T2 Single Shot sequences that are minimally sensitive to motion artifacts, along with a DWI respiratory-triggered sequence, as well as a minimally invasive sedation protocol. The data for this are currently under study and will be the subject of a future paper.

In our diagnostic approach for suspected urinary tract infection (UTI), we employed urinary dipstick testing, a method endorsed by recent literature that supports its use even in children younger than 2 years (3). Most urinary dipsticks in our study yielded positive results for a combination of nitrites, leukocyte esterase (LE), and red blood cells (RBC), while nearly one-third of samples were positive for LE and RBC without nitrites. Notably, we detected RBC in almost 90% of urinary dipsticks, and in 10% of cases, LE was present alone, despite positive urine cultures.

Urine culture results were used to make the definitive diagnosis of a UTI. However, we thoroughly evaluated each case using anamnestic, clinical, and laboratory data, including fever, leukocyturia, and bacteremia (2, 3). Even when the culture was contaminated with mixed flora, our assessment was based on a combination of urinary dipstick findings, clinical manifestations, ultrasound, and an examination that indicated a UTI.

Importantly, no statistically significant differences were observed in the characteristics of urinary dipsticks between patients with positive and negative MRI results, consistent with our previous study (23). Also, no disparities were noted between the implicated pathogen and the DWI result. In our case series, Escherichia coli (E. coli) was the most frequently identified microorganism in urine cultures (90.7%), slightly deviating from the epidemiological data outlined in Italian recommendations 2019 (3). Nonetheless, our findings closely align with other literature reports (1).

Upon examination of the enrolled patients, we did not observe significant demographic characteristics (such as age, weight, length, or gender) that distinguished positive from negative MRI results. This finding contrasts with a previous study, where patients with positive diffusion-weighted imaging (DWI) had a statistically different mean age of approximately 6 years compared to negatives, who had a mean age of 0.5 years (*p* = 0.004) (23). The higher rate of negative DWI exams in the previous study may explain this discrepancy.

Also, none of the patients in our study reported abdominal/lumbar pain or urinary symptoms because of their young age. In very young children, clinical manifestations of febrile urinary tract infection (fUTI) are nonspecific and may include fever, vomiting, diarrhea, reduced food intake, and irritability (1, 3, 35, 36). Also, 9.1% of our patients were admitted to the emergency room because of a rapid rise in fever and associated symptoms such as convulsions, hypotonia, loss of consciousness, and apnea with cyanosis. The literature reports that UTI could be a risk factor for febrile seizure (37), and newborns and infants may present with lethargy, irritability, apnea, and seizures (1).

A reduced percentage of patients exhibited foul-smelling urine, which, according to the literature, is neither specific nor sensitive enough to aid in the diagnosis of fUTI (3).

The clinical characteristics examined did not yield statistically significant differences, except for fever, who reaches the cut-off of the significance threshold, which was approximately 0.5°C higher in negative DWI cases compared with positive cases (39.5 (SD 0.5°C) vs. 38.9°C (SD 0.7), *p* = 0.05). However, because of the low number of negative cases, we consider this difference clinically irrelevant in distinguishing positives from negatives. This finding contrasts with our previous study, where positive patients had similar temperatures to negatives, but the number of negative cases was half that of positive cases (17 vs. 34) (23).

Previous studies did not find a statistically significant difference in temperature ranges between 38 and 40°C and renal parenchymal involvement (38). However, patients with positive DWI had a longer total duration of fever, which could indicate a delayed presentation to the emergency room and, therefore, a delayed initiation of antibiotic therapy. Although not statistically significant (*p* = 0.06), the hours of fever in positive cases were nearly double those in negative cases (∼85.5 vs. ∼44.3). In our previous research, we did not investigate this finding (23). It is necessary to confirm these results in a larger sample size study because they may be crucial in developing different strategies for the early diagnosis and treatment of UTIs in infants who do not have a specific febrile focus.

No studies in the literature compared the total duration of fever before MRI execution in our study's age range. However, some studies have shown significant differences in renal involvement in DMSA-Scintigraphy in acute patients with longer fever duration before hospital admission (38, 39). Also, similar studies conducted using DMSA have indicated that children treated on day 4 had an increased risk of acute APN compared to those treated within the first 24 h (40). In a prospective study of 278 infants with their first fUTI, initiating treatment within the first 24 h reduced the likelihood of acute APN (41).

In our study, all patients diagnosed with vesicoureteral reflux (VUR) (11/84) had positive DWI. Conversely, none of the patients with negative DWI presented VUR. VUR is historically associated with an increased risk of developing APN, but it does not represent a necessary or sufficient factor for acute renal damage in fUTIs, as most APN occurs in the absence of VUR (5, 38, 42, 43). In the literature, we did not find studies investigating VUR and acute renal parenchymal involvement in DWI in pediatric APNs; although, in young children, VUR is considered a risk factor for recurrent UTI and acquired renal scarring (44).

Between patients with positive DWI and the few with negative DWI, we found minimal differences in blood tests. We found no difference regarding BUN, creatinine, and eGFR; on the other hand, in literature, they are not indicated as routine tests to be performed in APNs, but they are considered more useful in monitoring the consequences of APNs such as scars (45, 46).

Regarding C-reactive protein (CRP) and procalcitonin (PCT), both showed a higher mean value in patients with positive DWI compared to negatives (7.7 (SD 6.7) vs. 3.9 (SD 4.8) mg/dl for CRP and 4.2(SD 13.8) vs. 2.3(SD 4.7) ng/ml for PCT), although the differences did not reach statistical significance. Notably, the standard deviations were particularly wide in both tests, for negatives and positives. These findings are consistent with our previous work, where CRP did not significantly differ between positives and negatives, while PCT was not evaluated (23).

Literature on this topic presents heterogeneous data. In the review by Shaik et al., PCT appears more promising for diagnosing APNs, yet the limited number of studies and marked heterogeneity among them restrict definitive conclusions. Consequently, routine use of either test in clinical practice is not recommended (9). However, the systematic review and meta-analysis conducted by Boon et al., involving 628 patients between 2 months and 18 years diagnosed with APN using DMSA, suggested that in children with signs suggestive of fUTI, a CRP level <2 mg/dl might aid in ruling out APN, while a PCT level ≥2 ng/ml might help in ruling it in (47). Also, a study by Pecile et al., not included in the Boon et al. meta-analysis, found that mean CRP levels were 10.6 (SD 6.9) mg/dl in patients with APN diagnosed with DMSA compared to 3.6 (SD 2.6) mg/dl in patients without renal involvement, while the mean PCT level was significantly higher in acute APN than in UTI without renal lesions (39).

These findings align with the Canadian review conducted by Leung et al. in 2019, where children exhibiting very high serum procalcitonin (PCT) levels during UTI were more likely to have acute APN. However, because of the considerable heterogeneity among studies, we must conduct further robust investigations before we can routinely recommend this test in the evaluation and management of pediatric UTI (1).

In our study, white blood cell count (WBC) was statistically higher in patients with positive DWI compared to negatives [15,736 (SD 5,944) vs. 10,531 (SD 3,063), *p* = 0.04]. Similarly, neutrophil counts were elevated in DWI-positive patients [9,270 (SD 4,843) vs. 6,553 (SD 2,847)], although the difference did not reach statistical significance. Notably, a significant increase in lymphocyte count was observed (*p* = 0.01) in patients with positive DWI.

Traditionally, lymphocytosis is not commonly associated with bacterial infections. However, the literature provides insights into the cellular and humoral events following experimental acute APN induced by P-fimbriated Escherichia coli. This study elucidates the relationship between cytokine increase, lymphocytes, and the response to bacterial infection, indicating that the inflammatory response is regulated not only by cytokine activity but also by lymphocyte activation (48). The clinical significance of these findings remains uncertain, and it is imperative to increase the number of negative cases to better contextualize this data. In our previous study, leukocytosis did not reach statistical significance, but data on neutrophils and lymphocytes were not reported (23).

Indeed, Pecile et al. in 2009 observed similar trends regarding leukocytosis. Their study indicated that children with white blood cell (WBC) counts exceeding 15,000 were 1.8 times more likely to exhibit positive DMSA-scan results compared to those with WBC counts below 11,000 cells (38).

In broader examinations encompassing larger studies and reviews, Boon et al. in 2021 underscored that blood markers such as WBC and neutrophils are deemed less reliable for diagnosing APN (48). Also, Leung in 2019 specified that while WBC and neutrophil counts exhibit low specificity and cannot accurately discriminate lower urinary tract infections from APN, neutrophilia is considered suggestive of APN (1).

Point of Strength of the study: our paper is the first cross-sectional study to evaluate renal parenchymal involvement with DW-MRI in children with their first episode of fUTI with a large sample of a homogeneous population cohort. There is no need for radiation or a contrast agent to detect parenchymal involvement. Midazolam combined with intranasal dexmedetomidine resulted in high family compliance in short-duration MRI examinations. To our knowledge, it is the only study with such population characteristics that compares inter-reader agreement between blinded radiologists in the reading of DW-MRIs.

Limitations of the study: the results obtained revealed a notable limitation stemming from the relatively small number of negative patients compared to positives, thereby compromising the statistical robustness of certain analyses. Also, the limited representation of negative cases in the MRI cohort precluded the calculation of threshold values for significant parameters using ROC curves. Also, the timing of DW-MRI varied, with examinations conducted between 24 and 72 h after admission. Consequently, patients underwent DWI following variable doses of antibiotics, potentially influencing the imaging outcomes. Moreover, logistical challenges inherent to sampling in young patients led to approximately half of the cohort undergoing only one urinary stick assessment. This constraint underscores the practical difficulties encountered in obtaining comprehensive urinary data in this age group.

In conclusion, DW-MRI emerges as a valuable tool for assessing renal parenchymal involvement in cases of fUTIs, showcasing substantial agreement among expert radiologists in blind evaluations, thereby affirming the reliability of this imaging modality. Our study revealed a limited correlation between clinical and laboratory parameters and DW-MRI results.

## Data Availability

The raw data supporting the conclusions of this article will be made available by the authors, without undue reservation.
